# Genome assemblies of two species of porcelain crab, *Petrolisthes cinctipes* and *Petrolisthes manimaculis* (Anomura: Porcellanidae)

**DOI:** 10.1093/g3journal/jkad281

**Published:** 2023-12-11

**Authors:** Pascal Angst, Eric Dexter, Jonathon H Stillman

**Affiliations:** Department of Environmental Sciences, Zoology, University of Basel, 4051 Basel, Switzerland; Department of Environmental Sciences, Zoology, University of Basel, 4051 Basel, Switzerland; Department of Environmental Sciences, Zoology, University of Basel, 4051 Basel, Switzerland; Department of Biology, San Francisco State University, San Francisco, CA 94132, USA; Department of Integrative Biology, University of California Berkeley, Berkeley, CA 94720, USA

**Keywords:** genomics, pacBio HiFi, genome assembly, Arthropoda, Crustacea, Decapoda, Malacostraca

## Abstract

Crabs are a large subtaxon of the Arthropoda, the most diverse and species-rich metazoan group. Several outstanding questions remain regarding crab diversification, including about the genomic capacitors of physiological and morphological adaptation, that cannot be answered with available genomic resources. Physiologically and ecologically diverse Anomuran porcelain crabs offer a valuable model for investigating these questions and hence genomic resources of these crabs would be particularly useful. Here, we present the first two genome assemblies of congeneric and sympatric Anomuran porcelain crabs, *Petrolisthes cinctipes* and *Petrolisthes manimaculis* from different microhabitats. Pacific Biosciences high-fidelity sequencing led to genome assemblies of 1.5 and 0.9 Gb, with N50s of 706.7 and 218.9 Kb, respectively. Their assembly length difference can largely be attributed to the different levels of interspersed repeats in their assemblies: The larger genome of *P. cinctipes* has more repeats (1.12 Gb) than the smaller genome of *P. manimaculis* (0.54 Gb). For obtaining high-quality annotations of 44,543 and 40,315 protein-coding genes in *P. cinctipes* and *P. manimaculis*, respectively, we used RNA-seq as part of a larger annotation pipeline. Contrarily to the large-scale differences in repeat content, divergence levels between the two species as estimated from orthologous protein-coding genes are moderate. These two high-quality genome assemblies allow future studies to examine the role of environmental regulation of gene expression in the two focal species to better understand physiological response to climate change, and provide the foundation for studies in fine-scale genome evolution and diversification of crabs.

## Introduction

Arthropoda is the largest and most diverse metazoan phylum (Thomas *et al*. 2020). Yet questions of genome evolution and diversification are limited to a relatively small number of clades (i.e. Diptera, Hymenoptera) for which a wealth of genome sequence data are available (Thomas *et al.* 2020). The crustaceans are one of the most diverse arthropod groups in terms of variation in morphology, habitat, and lifestyle, but also one of the most poorly represented arthropod groups in terms of whole genome sequence data. Of the six classes of Crustacea, the largest group, Malacostraca, contains about 40K species including crabs, shrimps, lobsters, crayfish, krill, amphipods, and isopods. Of the 3,750 complete Arthropod genomes currently available in the NCBI database of sequenced genomes, only 51 (∼1%) are for Malacostracans. If we consider only the Malacostracan order Decapoda, we observe tremendous species richness and diversity. The Decapoda contains over 14K extant species, of which the majority are the nearly 9K species of crabs (6.5K species of Brachyura and 2.4K species of Anomura, which includes the king, hermit, porcelain, and galatheid crabs) (De Grave *et al.* 2009). Despite the species richness and diversity of crabs, there are only 13 crab genome records in the NCBI database: 10 Brachyura, 5 of which are for the commercially important swimming crabs (Portunidae: *Portunus* and *Callinectes*), 4 of which are for the commercially important Chinese mitten crab (Varunidae: *Eriochir*), and 1 spider crab (Majidae: *Chionecetes*), and 3 Anomura (1 Coenobitidae: *Birgus*, 2 Lithodidae: *Paralithodes*). Clearly, if we are to understand the genomic basis of the ecological, physiological, and taxonomic diversification of such large and diverse group of arthropods, there is a need to develop better genomic resources for the Malacostraca, and for crabs in particular.

In this study, we produced functionally annotated long-read genome assemblies for two species of Anomuran porcelain crabs: *P. cinctipes* and *P. manimaculis.* Porcelain crabs, family Porcellanidae, are a species-rich group that inhabit shallow coastal ecosystems throughout the temperate and tropical regions of the Pacific Rim and warm regions of the Western Atlantic (Kropp and Haig 1994; Stillman and Reeb 2001; Raso *et al.* 2005; Rodriguez *et al.* 2005; Naderloo *et al.* 2013; Werding and Hiller 2015; Limviriyakul *et al.* 2016; Diez and Lira 2017; DE Azevedo Ferreira and Anker 2021; Mantelatto *et al.* 2021). The largest genus of Porcellanidae is *Petrolisthes* (Haig 1960). In the eastern Pacific, there are approximately 50 species of *Petrolisthes* split into four principal regions, the north temperate, the northern Gulf of California, the tropics, and the south temperate (Haig 1960; Stillman and Reeb 2001). Within each biogeographic region, species are distributed across vertical distribution gradients such that some species live solely in the intertidal zone and some are subtidal (Stillman and Somero 2000). As intertidal zone species are exposed to terrestrial conditions during low tide, they experience a wider range of environmental variation than subtidal zone species (Stillman 2002; Gunderson *et al.* 2019). Intertidal zone species possess physiological and morphological adaptations that allow them to survive the challenges of life out of water, including thermal variation and respiratory challenges (Stillman and Somero 1996; Gaitan-Espitia *et al.* 2014).

A molecular phylogenetic analysis of the eastern Pacific *Petrolisthes* indicated that there are two main subgenera or clades, which can be identified by the presence or absence of serrate saw-teeth on the meral segment of the chelae (Stillman and Reeb 2001). The clade possessing the serrate teeth is comprised mainly of species that live in tropical subtidal habitats; of the ∼25 species in that clade only two species have radiated to a different habitat: *Petrolisthes armatus*, inhabits tropical intertidal habitats, and *Petrolisthes desmarestii*, inhabits temperate subtidal habitats (Stillman and Reeb 2001). Thus, that clade has not had much adaptive radiation. Additionally, from phylogenetic analyses, the speciation events in the serrate teeth clade are well resolved (Stillman and Reeb 2001). In contrast, the other clade has an unresolvable polytomy at the base of the phylogenetic tree, with species that have radiated into every possible habitat (temperate, tropical, intertidal, subtidal) and evolved additional life-history innovations (e.g. specific commensalism). Only more recent speciation events within that clade are phylogenetically resolvable, and include additional radiation into different vertical zones within a biogeographic region (Stillman and Reeb 2001).

**Fig. 1.**
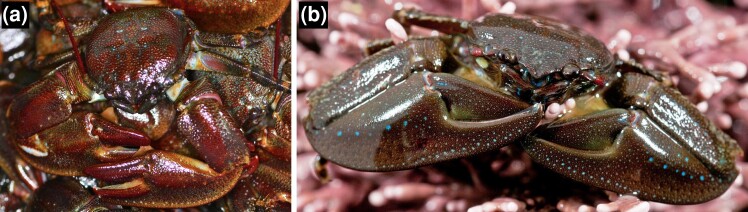
*Petrolisthes cinctipes* (a) and *Petrolisthes manimaculis* (b). Identifying marks on *P. cinctipes* include red antennae, red spots on claws, and red mouthparts. Identifying marks on *P. manimaculis* include lines of blue spots on claws, blue mouthparts, and red spots on base of gray antennae. *P. cinctipes* photograph by Adam Paganini and *P. manimaculis* photograph by Steven Sharnoff; both used with permissions.

In one of those subclades there are two sympatric species, *P. cinctipes* and *P. manimaculis* (Fig. 1), that share a common ancestor approximately 8–14 mya (Stillman and Reeb 2001), and live in different vertical zones on shores of the northeastern Pacific (Miller *et al.* 2013; Delmanowski and Tsukimura 2015; Armstrong and Stillman 2016; Delmanowski *et al.* 2017; Gunderson *et al.* 2017). These two species differ in their heat tolerance (Stillman and Somero 2000; Stillman 2002; Miller *et al.* 2013), and their responses to stress at the organismal (Wasson *et al.* 2002; Gunderson *et al.* 2017) and transcriptomic (Armstrong and Stillman 2016) levels. The genomic bases for the physiological differences between *P. cinctipes* and *P. manimaculis* are unknown.

Previous comparative studies of mitochondrial genomes have indicated that genome arrangements have likely played a strong role in the evolution of crabs (Wang *et al.* 2021; Zhang *et al.* 2021; Sun *et al.* 2022), and gene arrangement is known to be essential for emergent properties of gene products in development such as HOX genes (Sun and Patel 2019) and in cancer (Heng and Heng 2021, 2022). Evidence for the extent to which arrangement of nuclear genes is in general involved in adaptative evolution does not yet exist for crabs, but has been observed in other taxa including bacteria (Kang *et al.* 2022), fungal pathogens (Gourlie *et al.* 2022; Ma *et al.* 2022), and domesticated yeast (Garcia-Rios and Guillamon 2022), and may represent a generalized aspect of genome evolution during adaptive radiation (Cao *et al.* 2022; Wang *et al*. 2022). By providing two high-quality porcelain crab genome assemblies generated using long-read high-fidelity (PacBio HiFi) whole genome sequencing along with RNA-seq data for both species for genome annotation, we set the stage for exploring the extent to which differences in the physiology and ecology of *P. cinctipes* and *P. manimaculis* are reflected in their genomes.

## Methods

### Specimen collection, DNA extraction and sequencing

We produced de-novo genome assemblies *P. cinctipes* and *P. manimaculis* based upon Pacific Biosciences high-fidelity (PacBio HiFi) sequence data, which was then cross-validated and contaminant-filtered using independently generated cDNA library data, with additional 10× Genomics short-read data available from another study (J. Stillman, unpublished) for cross-validation in the case of *P. cinctipes*. PacBio sequencing was conducted using the HiFi method on gill tissues dissected from a single male crab specimen of each species collected near Fort Ross, California, USA (38.50421°N, 123.23152°W) on January 16, 2022 and frozen on liquid nitrogen and stored at −80 °C. Gill tissue was used because other tissue types did not provide suitable DNA for PacBio sequencing. Special attention was paid to bioinformatic filtering of nontarget DNA, because gill likely had a high load of epi-microbiota. Frozen tissues were delivered to UC Davis Genome Center DNA Technologies Core for high molecular weight (HMW) DNA extraction that yielded a fragment size peak of 155 and 132 Kb for *P. manimaculis* and *P. cinctipes*, respectively. PacBio HiFi libraries were prepared from those samples and each library was sequenced across three SMRT cells on the PacBio Sequel II platform.

10× Genomics sequencing was conducted on claw muscle tissue dissected from a single specimen of *P. cinctipes*. The specimen was likely collected at the site near Fort Ross, CA, USA (as above), but collection date of the specimen and the sex of the specimen are unknown. The HMW DNA extraction from claw muscle tissue is less prone to contamination and yielded adequate DNA of >40 Kb for 10× Genomics library construction. Libraries were size selected to 350–650 bp to maximize the quality of the paired end reads. Samples were sent to Novogene (Sacramento, CA, USA) for 150 bp PE sequencing on the Illumina HiSeqX10 platform.

RNA-seq data used in the analysis was obtained from Illumina 100 bp PE reads of cDNA libraries made from gill tissue of *P. cinctipes* and *P. manimaculis* as previously described (Armstrong and Stillman 2016). Additional transcriptomics data from ESTs of a cloned cDNA library of *P. cinctipes* are available (Tagmount *et al.* 2010), though not used in the present study.

### Assembly

**Fig. 2.**
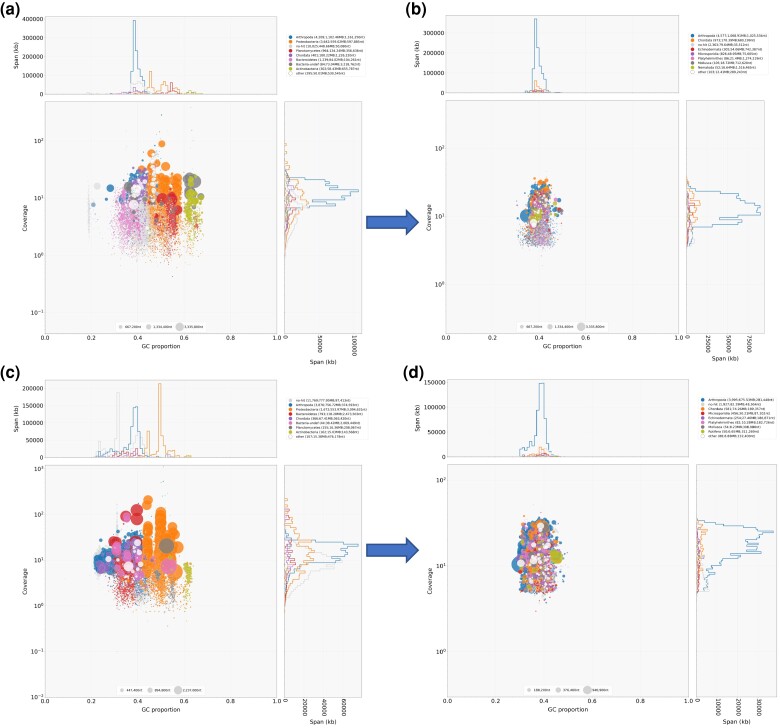
Filtering of raw sequence data from (a, b) *P. cinctipes* and (c, d) *P. manimaculis* using BlobTools. For each species, the coverage and GC content are compared (main plot *Y*- and *X*-axes), and are also plotted against sequence span for (a, c) unfiltered sequence data and (b, c) filtered sequence data. For both species, the effectiveness of sequence filtering can be observed by the enrichment of data represented by the blue Arthropod color in plots of sequence span vs GC proportion (top plot) and in plots of coverage vs sequence span (right plot).

Our primary genome assemblies were created using hifiasm v.0.16.0-r369 (Cheng *et al.* 2021). As our tissue samples likely contained nontarget DNA from epibionts and associated microbiota, our primary assemblies were carefully filtered to remove nontarget contigs. Contig filtering was performed using BlobTools v.1.1.1 (Laetsch and Blaxter 2017), which combines information about GC content, sequencing depth, and taxonomic classification to create a profile of each contig (Fig. 2). Based upon an iterative filtering process, we found that the following parameters removed all clearly nontarget contigs: GC content between 0.3 and 0.5 (five standard deviations from the mean of all unambiguously arthropod contigs) and sequencing depth between 0.33 and 3 times the average sequencing depth of all unambiguously arthropod contigs. We also removed circular contigs and contigs with strong sequence similarity to taxa outside the animal kingdom, except microsporidia, which tend to be wrongly annotated in reference databases likely because of tight host–parasite relations (intracellular parasitism). Sequencing depth for HiFi reads (required for BlobTools analysis) was calculated based on minimap2 map-hifi v.2.20-r1061 (Li 2018) mapping, while nucleotide alignments to reference databases (also required for BlobTools analysis) were done using blastn v.2.12.0+ (Camacho *et al.* 2009) and the NCBI database, as well as Diamond blastx -F 15 -b4 -c1 v.2.0.15.153 (Buchfink *et al.* 2021) and the UniProt database (The UniProt Consortium 2022). For *P. cinctipes*, we additionally filtered PacBio-based contigs based on independently generated 10× Genomics short-read data. We retained only contigs with mean 10× Genomics read depth between 1 and 286 (three times the mean whole-genome depth). 10× Genomics reads were mapped to the PacBio-based contigs using bwa-mem2 v.2.2.1 (Vasimuddin *et al.* 2019). For *P. manimaculis*, we additionally removed a set of contigs with no taxonomic classification which were responsible for an odd peak in the GC distribution around 0.33, likely arising from an unknown epibiont that was not represented in our BLAST database (Fig. 2).

BlobTools analyses indicated that 20% of the primary assemblies’ contigs had sequence similarity to Arthopoda, and the bulk of the sequences had either no strong similarity to any taxa (“No-hit”) or were microbial in origin (Table 1, Fig. 2). Following filtration of the data with BlobTools, the overall contig number was reduced by approximately 60% for both species, and the fraction of contigs with sequence similarity to Arthropoda increased to ∼50% for both species (Table 1). In fact, 75% or greater of the contigs in both species had similarity to either Arthropoda or had no similarity to any known taxa (Table 1, Fig. 2). Because further filtration of the data with BlobTools may have caused inadvertent discarding of *Petrolisthes* contigs, we kept all the contigs in the further analyses. Both final assemblies had similar proportions of taxonomic classification (Table 1).

**Table 1.** Assembly statistics before and after filtering the data using BlobTools.

Prefiltering			Postfiltering		
Taxon	# of contigs	%	Taxon	# of contigs	%
*Petrolisthes cinctipes*					
Arthropoda	5,361	42	Arthropoda	4,577	72
Proteobacteria	3,824	22	Chordata	973	11
Chordata	1,175	7	No-hit	2,364	5
No-hit	5,097	6	Echinodermata	305	4
Planctomycetes	1,144	5	Microsporidia	826	3
Bacteroidetes	1,579	4	Platyhelmithes	86	1
Bacteria-undef	97	3	Mollusca	105	1
Actinobacteria	342	2	Nematoda	52	1
Other	2,542	9	Other	103	<1
Total	21,161	100	Total	9,391	100
*Petrolisthes manimaculis*					
Arthropoda	5,214	35	Arthropoda	3,991	73
Proteobacteria	1,815	24	No-hit	1,926	9
No-hit	7,728	19	Chordata	581	8
Bacteriodetes	865	5	Microsporidia	456	3
Chordata	870	4	Echinodermata	254	3
Microsporidia	936	2	Platyhelmithes	82	1
Echinodermata	384	2	Mollusca	54	1
Rotifera	155	2	Rotifera	50	1
Other	977	6	Other	88	<1
Total	18,944	100	Total	7,482	100

Percentages refer to the sequence content per taxon.

All contig filtering was performed using seqtk v.1.3-r106 (https://github.com/lh3/seqtk), with before and after kmer distributions visualized using jellyfish v.2.2.10 (Marçais and Kingsford 2011) and GenomeScope v.2.0 (Ranallo-Benavidez *et al.* 2020). We used the default parameters for all bioinformatics tools if not mentioned otherwise.

### Annotation

After removal of all identifiable nontarget contigs in the assemblies, we masked repeats with lower-case letters using RepeatModeler v.2.0.2, including the LTR pipeline (Flynn *et al.* 2020), and ReapeatMasker v.4.1.2 (Smit *et al.* 2013). Masking the repeats using lower-case letters ensured that software for gene prediction was aware of them. We then performed quality and adapter trimming on the RNA-seq reads using trim_galore v.0.6.4_dev (https://github.com/FelixKrueger/TrimGalore) and cutadapt v.2.3 (Martin 2011) before mapping them to the respective species’ genome using HISAT2 v.2.2.1 (Kim *et al.* 2019). With the mapped reads, we trained GeneMark-ES v.4.62 (Brůna *et al.* 2020) and AUGUSTUS v.3.4.0 (Stanke et al. 2006, 2008) for gene prediction as implemented in BRAKER v.2.1.6 (Brůna *et al.* 2021). The resulting annotation files were converted to GFF3 files and to sequence files using AGAT v.1.0.0 (Dainat 2022). The resulting files were used for functional annotation with the combined evidence from InterProScan v.5.55_88.0 (Jones *et al.* 2014), eggNOG-mapper v.2.1.9 (Huerta-Cepas *et al.* 2019; Cantalapiedra *et al.* 2021), Phobius v.1.01 (Käll *et al.* 2004), and SignalP v.5.0b (Almagro Armenteros *et al.* 2019) as well as with comparisons to Pfam (Mistry *et al.* 2021), UniProt (The UniProt Consortium 2022), MEROPS (Rawlings *et al.* 2014), dbCAN (Yin *et al.* 2012) databases, and BUSCO (Manni *et al.* 2021) with the Arthropoda database (arthropoda_odb10) with funannotate 1.8.11 (Palmer and Stajich 2022). Annotation statistics were generated with agat_sp_statistics.pl v.1.0.0 (Dainat 2022).

### Comparative genomics

We examined synteny between the two species in terms of sequence homology across large contigs and in terms of the order of orthologous gene pairs. For assessing sequence similarity on the contig level, we used D-Genies v.1.5.0 (Cabanettes and Klopp 2018). For gene level comparisons, we only used single copy orthologs inferred with OrthoFinder v.2.5.4 (Emms and Kelly 2019). Each information was used to identify 10 homologous contigs for in-depth sequence comparison (Supplementary Fig. 1). For visualization of the syntenic regions, we used GENESPACE v.1.1.7 (Lovell *et al.* 2022) and gggenomes v.0.9.5.9000 (Hackl *et al.* 2021) in R v.4.2.2 (R Core Team 2022). To estimate divergence between single copy orthologs, we aligned them using prank v.170427 (Löytynoja 2014) while using seqinR v.4.2–23 (Charif and Lobry 2007) for file handling. The alignment was followed by a masking step, in which poorly aligned sequences were excluded from downstream analysis. Sequence divergence was then calculated using CodeML of the paml v.4.9 package (Yang 2007). A phylogenetic tree based on available Anomuran and Brachyuran crab genomes was inferred using IQ-TREE2 v.2.1.4-beta (Minh *et al.* 2020) as implemented in funannotate's compare function with the spiny lobster, *Panulirus ornatus* (Veldsman *et al.* 2021), as an outgroup. For this, we performed 1,000 bootstrap replicates.

## Results

PacBio HiFi sequencing resulted in a total throughput of 63.1 Gb for *P. cinctipes* (read N50: 12.9 Kb) and 81.4 Gb for *P. manimaculis* (read N50: 13.6 Kb), which we individually used for genome assembly of the two species. The assembled and filtered genome for *P. cinctipes* comprised 9.4K contigs with an assembly N50 of 707 Kb and a total length of 1.49 Gb (Table 2). The number of contigs (7.5K), assembly N50 (219 Kb), and total length (0.92 Gb) were all lower for *P. manimaculis* (Table 2). The genome assembly length of *P. cinctipes* was closer to the genome size estimate of a species of the same genus (∼2.05 Gbp in *P. galathinus*; Rheinsmith *et al.* 1974). Despite the differences in overall sequence length, the two genomes had equivalent completeness with 94% and 95% complete BUSCOs in *P. cinctipes* and *P. manimaculis*, respectively (Table 2 and Supplementary Table 1). Additionally, the total number of protein-coding genes identified was similar in the two species, with 45K and 40K for *P. cinctipes* and *P. manimaculis*, respectively (Table 2). Within protein coding genes, the mean transcript length, exon length, and exons per gene were also similar between the two species (Table 2).

**Table 2:** Sequencing and annotation statistics for two species of porcelain crab.

	*Petrolisthes cinctipes*	*Petrolisthes manimaculis*
*Assembly*		
Total length (Gb)	1.49	0.92
GC content (%)	39.63	38.23
Contig N50 (Kb)	706.73	218.94
Contig number	9391	7482
BUSCO completeness score (%)	91.7	92.3
*Annotation*		
Total length of repeats (Gb)	1.12	0.54
% Repeats	75%	59%
Number of protein-coding genes	44,543	40,315
Mean transcript length (bp)	6,672	6,277
Mean coding sequence length (bp)	1,200	1,218
Mean exon length (bp)	299	275
Mean intron length (bp)	1,362	1,188
Average exons per gene	4.8	5.1
BUSCO completeness score (%)	94.3	95.1

**Fig. 3.**
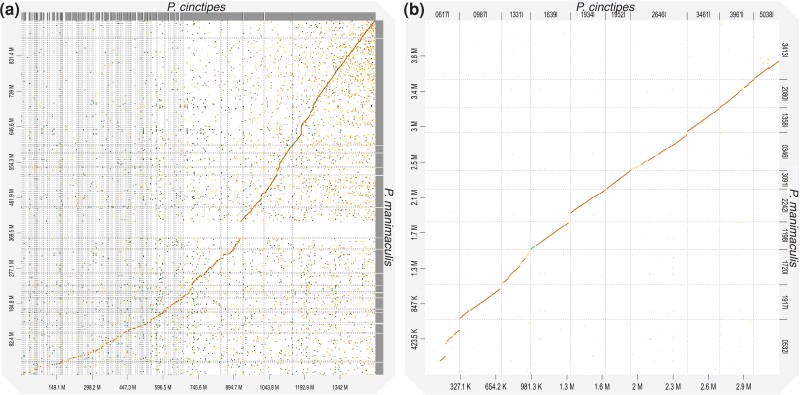
Pairwise dotplots for (a) entire genome assemblies and (b) 10 selected homologous contigs (also see Supplementary Fig. 1, Table 4). In both plots, contigs from *P. cinctipes* are on the *X*-axis and contigs from *P. manimaculis* are on the *Y*-axis. In panel a, what looks like a gap in the *P. cinctipes* assembly is an assortment of numerous small *P. manimaculis* contigs (clustered together by D-genies) not present in *P. cinctipes*. If the sorting and clustering would be done based on *P. manimaculis*, a gap-like pattern would appear in the *P. manimaculis* assembly.

**Fig. 4.**
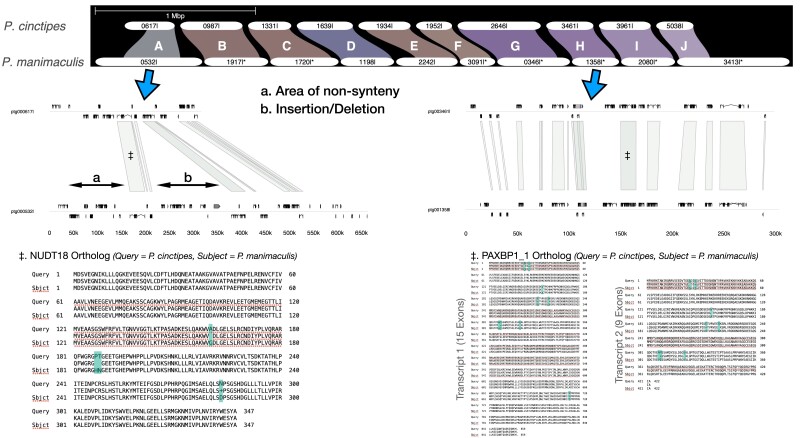
Syntenic map of orthologous regions among *P. cinctipes* and *P. manimaculis* for contigs selected based on gene density and size (see Fig. 3, Table 4). Ribbons are named (“A” to “J”) and color coded by contig. The beginning of each contig name (ptg00) was trimmed and contigs with an asterisk were inverted to improve visibility. Gene order and spacing for two contig pairs (“A” and “H”) are provided, and gene sequence comparison for one of the larger genes within each contig pair illustrates interspecific differences in the genomes at structural (e.g. nonsynteny and indel in “A”) and sequence levels (blue highlighted amino acids in sequences). For more information about each contig pair, see Table 4, and for detailed figures of each contig pair, see Supplementary Fig. 2.

Comparing the sequence content of the two genome assemblies, the difference in length can largely be explained by differing level of repeat content (Table 3). Despite the presence of many repetitive regions, which might be species specific, we found that 36.47% of the *P. cinctipes* sequence had matches in *P. manimaculis* by whole genome sequence alignment (Fig. 3a). To be able to compare genetic regions that not only share sequence similarity but also share the same evolutionary origin, i.e. are homologous, we used alignments of single copy orthologs. Assessing divergence between single copy orthologs of the two species, we found a mean *d*_S_ value of 0.154 (95% confidence interval 0.148–0.160) and a mean *d*_N_/*d*_S_ value of 0.266 (0.260–0.273) indicating a relatively low level of divergence. For a larger scale comparison of homologous sequence between the two species, we identified 10 contigs of at least 200 Kb in length in which the two species shared at least 17 single copy orthologs (Supplementary Fig. 1 and Fig. 3b). An examination of those contig pairs indicated that genes were always in a similar order (Fig. 4, top panel), though there were some differences between the genomic regions in terms of gene spacing (Fig. 4, middle panels) and gene sequence (Fig. 4, bottom panels). For example, in contig pair A, there is a region of insertion/deletion of approximately 150 Kb (Fig. 4, middle panels). Homologous contig pairs were for the most part syntenic in their overlapping regions (Table 4 and Supplementary Fig. 2), but in contig pair A, there was a nonsyntenic region in which none of the genes were shared between species (Fig. 4). Orthologs within these contig pairs showed very high sequence homology.

**Table 3.** Different repeat categories found using RepeatModeler coupled with RepeatMasker.

	*Petrolisthes cinctipes*	*Petrolisthes manimaculis*
Retroelements	203	100
DNA transposons	29	25
Unclassified	806	329
Small RNA	2	2
Satellites	<1	<1
Simple repeats	67	78
Low complexity	6	8
Total	1,115	543

All values are in Mb. Unclassified repeats could not be assigned to any category and might represent species-specific repeat families.

**Table 4.** Interspecific synteny analysis of contigs from *P. cinctipes* (Cinc) and *P. manimaculis* (Mani) selected on the basis of length and number of single copy orthologous genes (orthologs).

Contig pair	Cinc_Contig	Mani_Contig	Length of overlap (Kb)	Nr. shared single copy orthologs	Nr. Cinc nonsingle copy orthologs	Nr. Mani nonsingle copy orthologs	IDs Cinc nonsingle copy orthologs [Pcinc_v1.9_]	IDs Mani nonsingle copy orthologs [Pmani_v1.7_]
A	ptg000617l	ptg000532l	244	8	2	14	g782, g783	g3404, g3405, g3406, g3407, g3408, g3409, g3410, g3411, g3413, g3416, 4266_g, g3417, g3418, g3419
B	ptg000987l	ptg001917l	365	23	13	10	10939_g, g42538, g42539, g42540, g42544, 10944_g,, g42551, g42552, 10956_g, g42558, g42560, g42564, g42565	g29145, 15539_g, g29148, g29152, 15545_g, 15547_g, g29154, g29160, 15562_g, g29171
C	ptg001331l	ptg001720l	275	19	2	13	g1285, g1286	g19593, g19595, g19596, g19608, g19609, g19613, g19614, g19615, g19616, g19617, 14126_g, g19618, g19619
D	ptg001639l	ptg001198l	334	22	6	13	g34991, g34993, g35004, g35005, g35010, g35013	g16436, g16437, g16438, g16440, g16444, 10076_g, g16448, 10078_g, 10082_g, g16450, g16456, g16458, g16463
E	ptg001934l	ptg002242l	239	18	9	2	g3817, g3820, g3821, g3825, g3828, 18677_g, g3830, g3832, g3835	g31995, g31999
F	ptg001952l	ptg003091l	204	42	7	14	g17612, g17632, 18771_g, g17646, g17651, 18794_g, g17656	g14228, g14237, g14239, g14241, g14242, g14243, g14244, g14246, g14256, g14258, g14259, g14276, g14280, g14281
G	ptg002646l	ptg000346l	409	19	11	3	g16185, g16186, g16188, g16191, g16192, g16194, g16195, g16196, g16197, g16198, g16207	g4887, g4891, g4900
H	ptg003461l	ptg001358l	270	17	9	3	g39945, 28259_g, 28263_g, g39954, g39955, g39956, g39958, g39963, g39964	g28965, g28967, 11234_g
I	ptg003961l	ptg002080l	321	18	7	6	g22530, g22531, g22534, 31088_g, 31090_g, 31091_g, g22544	g8956, g8957, g8969, g8970, 16629_g, g8973
J	ptg005038l	ptg003413l	188	29	11	9	g29107, g29109, g29122, g29126, g29127, g29128, g29130, g29134, g29135, g29136, g29137	g1785, g1789, g1790, g1791, g1792, g1796, g1797, g1802, g1815

Ortholog IDs starting with “g” were predicted by AGUSTUS gene predictor software, and ortholog IDs ending with “_g” were predicted by GeneMark.hmm gene predictor software (see methods for details). Please see the full GFF file for additional details on each ortholog. Contig pairs refer to Fig. 4.

**Fig. 5.**
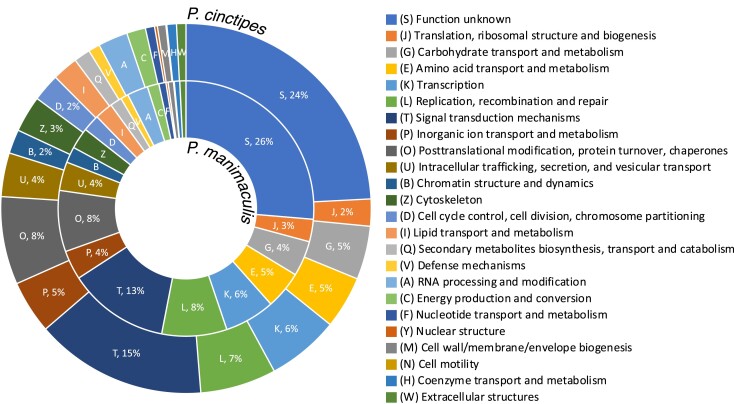
Predicted functions of gene sets based on clusters of orthologous genes (COGs). Depicted are the distributions of the predicted gene function categories in *P. cinctipes* (outer ring) and *P. manimaculis* (inner ring). These distributions are similar. The legend's order reflects the clockwise order of the functional categories in the graph. Categories which consist of only a few genes are not labeled with percentages to increase visibility.

**Fig. 6.**
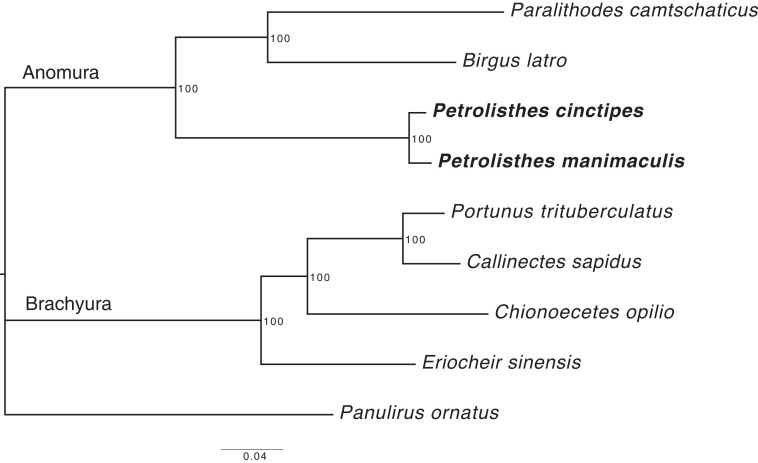
Maximum-likelihood tree of available Brachyuran and Anomuran genomes. The two *Petrolisthes* species are less related to the other two Anomuran crab species than these two other species to each other. The spiny lobster, *P. ornatus* (Palinuridae), was used as an outgroup. Node labels represent bootstrap values.

Focusing on the predicted gene functions obtained from funannotate, we found similar distributions in both *Petrolisthes* genomes (Fig. 5), which was expected for species of the same genus. The combined study of the crabs gene arrangement with their expression level will hopefully provide insight into their adaptive evolution. Using additional Anomuran and Brachyuran crab species’ genomes, available from the NCBI database (Table 5), we conducted a phylogenetic analysis. We identified 114 single-copy orthologs and generated a maximum-likelihood tree which supports the phylogenetic placement of the Porcellanidae within the Anomura separate from the Lithodidae (*Paralithodes camtschaticus*) and Coenobitidae (*Birgus latro*) (Fig. 6) (Wolfe *et al.* 2021).

**Table 5.** Summary of Brachyuran and Anomuran genome sequencing projects.

Species/(Infraorder)	Genome Size (Gb)	Repeat %	GC Content %	Protein coding genes (1,000 s)	Complete BUSCO %	Reference
Brachyura						
*Callinectes sapidus* (Portunidae)	1.1	36	40	25	93	Bachvaroff *et al*. (2021)
*Portunus trituberculatus* (Portunidae)	1.0	54	41	17	95	Tang *et al.* (2020)
*Eriochir sinensis* (Varunidae)	1.6	45	41	28	92	Cui *et al*. (2021)
*Chionecetes opilio* (Majidae)	2	NA	42	22	NA	NCBI GCA_016584305.1
Anomura						
*Paralithodes platypus* (Lithodidae)	4.8	78	42	28	77	Tang *et al*. (2021)
*Paralithodes camtschaticus* (Lithodidae)	7.3	68	41	29	90	Veldsman *et al*. (2021)
*Birgus latro* (Coenobitidae)	6.2	24	42	24	90	Veldsman *et al*. (2021)
*Petrolisthes cinctipes* (Porcellanidae)	1.5	75	40	45	94	This Study
*Petrolisthes manimaculis* (Porcellanidae)	0.9	59	38	40	95	This Study

## Discussion

Crabs are an exceptionally species-rich and diverse taxon (De Grave *et al.* 2009; Wang *et al.* 2021), whose evolution might be driven by ecology, physiology, and gene rearrangements (Tang *et al.* 2021; Veldsman *et al.* 2021; Wang *et al.* 2021). Available nuclear genomic resources of crabs are sparse but needed for comparative genomic approaches, which would allow investigating the evolutionary role of these factors in crabs. Here, we present two Anomuran crab genome assemblies, *P. cinctipes* and *P. manimaculis*, the first ones of the Anomuran porcelain crabs, family Porcellanidae. We found differences in genome size, genome structure, and gene sequence in homologous regions of the genomes. The largest differences between the two species include a larger genome size and a higher repeat content of *P. cinctipes*. Together, these findings suggest that minor differences in coding regions reflect just a part of the different evolutionary trajectories of the two species when considered with larger scale structure of the two species’ genomes.

Though there are about 9K crab species, genome sequence data are sparse: Only few nuclear genome assemblies (*n* = 13) are available, mainly for four species of Brachyuran (*n* = 10) crabs and three species of Anomuran (*n* = 3) crabs (Table 5). The genomes of Brachyuran crabs have been assembled to chromosome-level in Portunidae [*Callinectes sapidus “*blue crab” (Bachvaroff *et al.* 2021), *Portunus trituberculatus* “swimming crab” (Tang *et al.* 2020)] and Varunidae (*Eriocheir sinensis “*Chinese mitten crab”; Cui *et al.* 2021], as well as to a nonchromosome level in Majidae [*Chionecetes opilio “*snow crab” (NCBI database; Assembly name: ASM1658430v1; GenBank assembly accession: GCA_016584305.1; Bioproject accession: PRJNA602365)]. While not a chromosome-level assembly, the genome of a third Portunid crab has been sequenced and had a similar genome size and other characteristics to the other Portunid crabs (*Charybdis japonica “*Asian paddle crab”; Liu *et al.* 2022). Only one Anomuran crab species has been assembled to chromosome-level (Lithodidae: *Paralithodes platypus* “blue king crab”; Tang *et al.* 2021) and there are nonchromosomal genome assemblies for two additional species (Lithodidae: *Paralithodes camtschaticus* “red king crab” and Coenobitidae: *Birgus latro “*coconut crab”; Veldsman *et al.* 2021). Comparing our genome assemblies to the available ones, the GC content in all assemblies is close to 40%. Our assemblies feature the highest completeness measured as BUSCO score and the highest number of genes. Assembly lengths of our porcelain crab assemblies are more similar to the Brachyuran crabs than to the other Anomuran crabs, which have about four times longer assemblies. Our porcelain crab assemblies, however, have a repeat content amount that is more similar to other Anomuran crabs than to Brachyuran crabs. These differences might reflect differential evolution among crab species (Iannucci *et al.* 2022) but might also partially arise from sequencing artifacts owing to the different sequencing technologies used for the assemblies.

Previous studies on the two *Petrolisthes* species studied here have found that their responses to stress differ at the transcriptomic level (Armstrong and Stillman 2016). Given that gene arrangements have been suggested to be involved in the mitochondrial evolution of crabs (Wang *et al.* 2021; Zhang *et al.* 2021; Sun *et al.* 2022), the question arises of the extent to which differences in the physiology and ecology of the two crab species is reflected in their genomes. A common hypothesis is that under stress, there are more genetic rearrangements (Heng and Heng 2021, 2022). The high-quality genome assemblies of the two porcelain crab species presented here can be combined with existing knowledge of their ecological, physiological, and transcriptional differences to provide a maximally integrative investigation of their adaptive evolution and understanding of the mechanisms driving their physiological differences. For example, the absolute and relative location of differentially expressed genes in the two species can be compared, which allows inferences about their mobility level as compared to nondifferentially expressed genes. Such investigations should consider the genome size differences in the here-generated assemblies. The larger genome *P. cinctipes* features more repetitive regions (Table 3) and a higher number of duplicated BUSCO genes (Supplementary Table 1), which could indicate different performance of the applied software for assembly or biological differences. In the latter, relaxed selection could enable the proliferation of repetitive elements and gene duplication in *P. cinctipes*. The generated genome assemblies add to an ever-increasing number of available crab genomes, improving the potential for deeper insights into the evolution of the genomes and the diverse traits in Anomuran and Brachyuran Decapod crustaceans.

Comparative genomics yield most valuable insights when applied to completest possible genome assemblies of highest possible quality. Our genome assemblies were generated based on the most reliable available sequencing technology for de novo genome sequencing, but there are options which might improve their contiguity further. For example, with the available resources, it is possible to use the sequence information from one species to scaffold the genome assembly of the other species and vice versa, because of their moderate sequence divergence. This approach, however, would only improve the assemblies to a small degree and at the same time might lead to wrong sequence links in cases of genomic rearrangements. Other possibilities for scaffolding and therefore improving the assemblies would involve the generation of additional data using Oxford Nanopore (Price *et al.* 2023; Salson *et al.* 2023) and Hi-C (Bracewell *et al.* 2023) sequencing, both of which are established scaffolding approaches. Furthermore, even though we applied state-of-the-art methodology to identify (non-)focal DNA sequence in our assemblies, future work should focus on generating higher quality DNA from less contaminant-prone tissue, like muscle issue. This would reduce uncertainty in the identification of (non-)focal DNA sequence, i.e. reduce the number of nonfocal contigs still included in the genome assemblies. Using approaches such as those would be a next step toward generating the first-ever chromosome-level assembly for a porcelain crab.

## Supplementary Material

jkad281_Supplementary_Data

## Data Availability

Raw data is deposited at the NCBI SRA database, and the assembled genomes as well as the predicted sets of protein sequences are available at the NCBI GenBank database (BioProject ID: PRJNA1002960) and at https://doi.org/10.6084/m9.figshare.23823531. Analysis scripts are deposited at https://github.com/pascalangst/Petrolisthes_assemblies. Supplemental material available at G3 online.
