# Correction to: Genome assemblies of two species of porcelain crab, *Petrolisthes cinctipes* and *Petrolisthes manimaculis* (Anomura: Porcellanidae)

**DOI:** 10.1093/g3journal/jkae027

**Published:** 2024-02-20

This is a correction to: Pascal Angst, Eric Dexter, Jonathon H Stillman, Genome assemblies of two species of porcelain crab, *Petrolisthes cinctipes* and *Petrolisthes manimaculis* (Anomura: Porcellanidae), *G3 Genes|Genomes|Genetics*, Volume 14, Issue 2, February 2024, https://doi.org/10.1093/g3journal/jkad281

In the originally published version of this manuscript, in the legend for Figure 1 the accreditations for the images were transposed. These should read: “*P. cinctipes* photograph by Adam Paganini and *P. manimaculis* photograph by Steven Sharnoff; both used with permissions.” instead of: “*P. cinctipes* photograph by Steven Sharnoff and *P. manimaculis* photograph by Adam Paganini; both used with permissions.”

This error has been emended in the article.

