# Supplementary material for: Genome assemblies of two species of porcelain crab, Petrolisthes cinctipes and Petrolisthes manimaculis (Anomura: Porcellanidae)
Source: G3 (Bethesda). 2023 Dec 11;14(2):jkad281. doi: 10.1093/g3journal/jkad281 (PMC10849366; doi:10.1093/g3journal/jkad281)
Supplement: jkad281_Supplementary_Data [file jkad281_supplementary_data.docx]

**Supplemental Information for:**

**Genome assemblies of two species of porcelain crab, *Petrolisthes cinctipes* and *Petrolisthes manimaculis* (Anomura: Porcellanidae)**

Pascal Angst^1,*^, Eric Dexter^1^, Jonathon H. Stillman^1,2,3,*^

^1^ Department of Environmental Sciences, Zoology, University of Basel, Switzerland

^2^ Department of Integrative Biology, University of California Berkeley, USA

^3^ Department of Biology, San Francisco State University, USA

^*^ Corresponding authors: [pascal.angst@unibas.ch](mailto:pascal.angst@unibas.ch); [stillmaj@sfsu.edu](mailto:stillmaj@sfsu.edu)

**Table S1. BUSCO version 5.1.2 results using and arthropoda_odb10**

|  | *Petrolisthes cinctipes* | | *Petrolisthes manimaculis* | |
| --- | --- | --- | --- | --- |
|  | Number | Percent | Number | Percent |
| Complete BUSCOs (C) | 929 | 91.7 | 935 | 92.3 |
| Complete and single-copy BUSCOs (S) | 757 | 74.7 | 807 | 79.7 |
| Complete and duplicated BUSCOs (D) | 172 | 17 | 128 | 12.6 |
| Fragmented BUSCOs (F) | 32 | 3.2 | 33 | 3.3 |
| Missing BUSCOs (M) | 52 | 5.1 | 45 | 4.4 |
| Total BUSCO groups searched | 1013 |  | 1013 |  |

**Figure S1. Plot showing identification of the selected “optimal-set” contig pairs for synteny analyses from 2,377 total contigs containing a total of 7,008 orthologous genes (orthologs).** These contig pairs contain at least one shared ortholog and the contigs themselves are the best alignment between the two species genomes. The selection strategy returned the top 10 longest contigs of at least that had the greatest number of shared single-copy orthologs by rank-ordering along each axis.

**Figure S2 (A-J). Plots of orthologous contigs from *Petrolisthes cinctipes* (top contig) and *Petrolisthes manimaculis* (bottom contig).** Grey lines are drawn between orthologous genes between the two species. Black vertical lines within each contig represent gene exons and horizontal ^ shaped lines represent gene introns. Contig length in Kb is indicated on the bottom of each panel. See Figure S1 for identification of these contigs and Table 4 for additional details.

(A)

__

(B)

(C)

(D)

(E)

(F)

(G)

(H)

(I)

(J)
